# Supplementary material for: Performance Enhancement in Powder-Fabricated Cu2(ZnSn)Se4 Solar Cell by Roll Compression
Source: Materials (Basel). 2023 Jan 26;16(3):1076. doi: 10.3390/ma16031076 (PMC9921508; doi:10.3390/ma16031076)
Supplement: Supplementary file 1 [file materials-16-01076-s001.zip › materials-2039625-supplementary.pdf]

materials

Supplementary Information

Performance Enhancement in Powder-Fabricated $Cu_2(ZnSn)Se_4$ Solar Cell by Roll Compression

Jaehyun Park [1], Hyobin Nam [1], Bong-Geun Song [1], Darya Burak [1], Ho Seong Jang [1,2], Seung Yong Lee [1,2], So-Hye Cho [1,2,*] and Jong-Ku Park [1,*]

[1] Materials Architecturing Research Center, Korea Institute of Science and Technology, Hwarangno 14-gil 5, Seongbuk-gu, Seoul 02792, Republic of Korea
[2] Division of Nanoscience and Technology, KIST School, Korea University of Science and Technology, Seoul 02792, Republic of Korea
* Correspondence: sohyec@kist.re.kr (S.-H.C.); jkpark@kist.re.kr (J.-K.P.)

Citation: Park, J.; Nam, H.; Song, B.-G.; Burak, D.; Jang, H.S.; Lee, S.Y.; Cho, S.-H.; Park, J.-K. Performance Enhancement in Powder-Fabricated $Cu_2(ZnSn)Se_4$ Solar Cell by Roll Compression. *Materials* **2023**, *16*, 1076. https://doi.org/10.3390/ma16031076

Academic Editor: Eunsoon Oh

Received: 02 November 2022
Revised: 17 January 2023
Accepted: 22 January 2023
Published: 26 January 2023

Copyright: © 2023 by the authors. Licensee MDPI, Basel, Switzerland. This article is an open access article distributed under the terms and conditions of the Creative Commons Attribution (CC BY) license (https://creativecommons.org/licenses/by/4.0/).

(a)

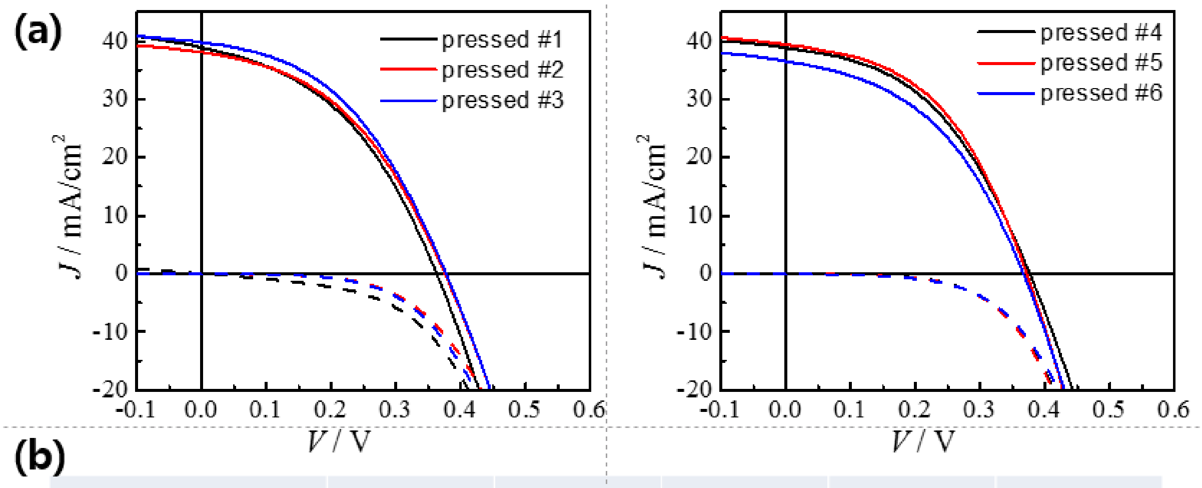

(b)

Device Name	Voc / V	Jsc / mA/cm²	FF / %	Eff / %	Area / cm²
pressed #1	0.362	38.877	42.084	5.923	0.414
pressed #2	0.375	38.091	42.946	6.134	0.431
pressed #3	0.377	39.733	43.369	6.497	0.399
pressed #4	0.376	38.850	44.579	6.512	0.422
pressed #5	0.372	39.392	46.511	6.816	0.400
pressed #6	0.366	36.544	43.990	5.884	0.437

Figure S1. (a) *j–V* curves and (b) device performance parameters of pressed CZTSe cells under AM 1.5G illumination (solid lines) and in the dark (dotted lines).

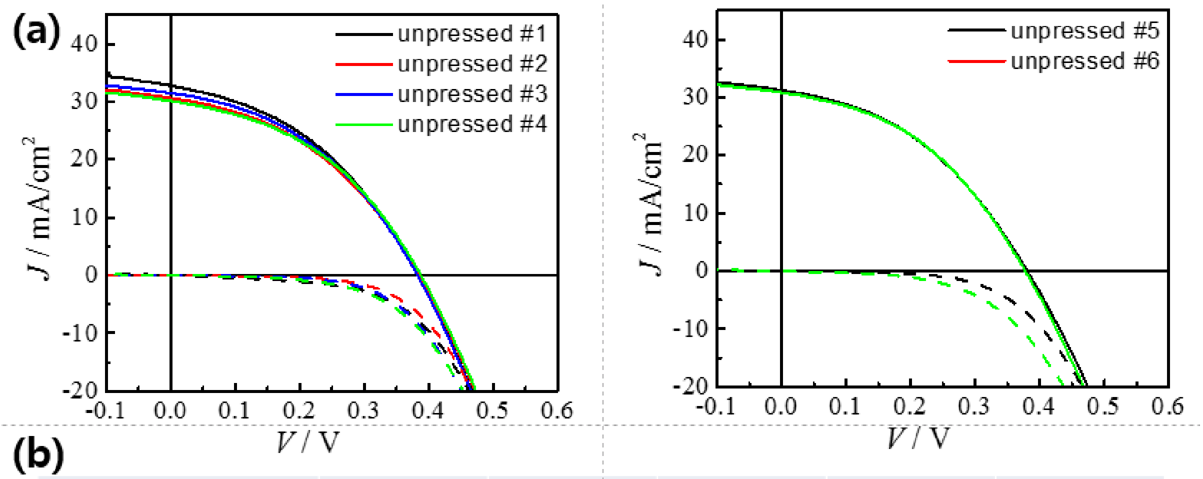

(b)

Device Name	Voc / V	Jsc / mA/cm²	FF / %	Eff / %	Area / cm²
unpressed #1	0.383	32.789	40.651	5.105	0.428
unpressed #2	0.386	30.620	40.847	4.828	0.444
unpressed #3	0.383	31.493	41.009	4.901	0.440
unpressed #4	0.388	30.135	41.924	4.902	0.434
unpressed #5	0.381	31.233	40.388	4.806	0.437
unpressed #6	0.377	30.827	41.449	4.817	0.433

Figure S2. (a) *j*–*V* curves and (b) device performance parameters of unpressed CZTSe cells under AM 1.5G illumination (solid lines) and in the dark (dotted lines).
